# Longitudinal cytokines in plasma extracellular vesicle enriched preparations and motor worsening in Parkinson disease

**DOI:** 10.3389/fimmu.2026.1973023

**Published:** 2026-10-21

**Authors:** Chien-Tai Hong, Yi-Chen Hsieh, Lung Chan, Wei-Ting Chiu, Chin-Hsuan Sophie Lin, Chen-Chih Chung

**Affiliations:** 1 Department of Neurology, Taipei Medical University-Shuang Ho Hospital, New Taipei, Taiwan; 2 Department of Neurology, School of Medicine, College of Medicine, Taipei Medical University, Taipei, Taiwan; 3 Ph.D. Program in Medical Neuroscience, College of Medical Science and Technology, Taipei Medical University, Taipei, Taiwan; 4 Melbourne School of Psychological Sciences, The University of Melbourne, Melbourne, VIC, Australia; 5 Melbourne Brain Centre for Imaging Unit, The University of Melbourne, Melbourne, VIC, Australia

**Keywords:** cytokines, extracellular vesicles, inflammation, longitudinal study, machine learning, Parkinson’s disease

## Abstract

**Introduction:**

Inflammation is implicated in Parkinson disease (PD), but the prognostic value of circulating cytokines beyond clinical information remains incompletely defined.

**Methods:**

We studied cytokines measured in polymer-precipitated plasma extracellular vesicle (EV)-enriched preparations in a longitudinal cohort of 62 participants with PD; 21 healthy controls contributed baseline descriptive data. Fifteen cytokines were assessed using multiplex immunoassays. Contemporaneous cytokine -clinical score associations were examined using covariate-adjusted generalized estimating equations (GEE) with false discovery rate (FDR) correction. Prediction analyses used 147 consecutive follow-up intervals, with worsening defined as an increase of at least three points in the original Unified Parkinson's Disease Rating Scale Part III. ON-medication status for motor examinations was confirmed by patient report; exact dose-to-assessment intervals were not recorded. Clinical-only, cytokine-only and combined radial basis function support vector machine (RBF-SVM) models were compared using participant-grouped nested cross-validation.

**Results:**

Eight of 105 cytokine-by-visit interactions met the FDR threshold after adjustment including baseline disease duration. Clinical-only, cytokine-only and combined pooled out-of-fold areas under the curve (AUCs) were 0.733, 0.722 and 0.784, respectively. The combined-minus-clinical AUC difference was 0.051 (95% confidence interval −0.009 to 0.112). At the five-point sensitivity threshold, the corresponding AUCs were 0.743, 0.675 and 0.790, with a difference of 0.047 (−0.023 to 0.112).

**Discussion:**

Cytokines in EV-enriched preparations showed visit-dependent clinical associations and numerically higher discrimination when combined with clinical predictors within the RBF-SVM framework. Independent validation and matched conventional plasma measurements are needed to determine clinical usefulness and preparation-specific value.

## Introduction

Parkinson’s disease (PD) is characterized by dopaminergic neurodegeneration and alpha-synuclein aggregation. Inflammation is implicated in PD pathobiology, with altered immune activity in the central nervous system and peripheral blood (1). These observations motivate investigation of accessible immune biomarkers that can be assessed alongside clinical outcomes.

Blood EV preparations have been investigated as sources of PD-related biomarkers (2–4). Cytokines can occur in association with EVs in experimental systems (5), but measurements in an isolated preparation do not by themselves establish cytokine localization. Polymer precipitation can co-enrich non-vesicular particles and soluble proteins. Terminology should therefore reflect the preparation and the characterization performed (6).

Previous studies have reported altered cytokine profiles in plasma EV preparations and associations with motor and cognitive outcomes in PD (7, 8). The longitudinal behavior of these measurements and their predictive value beyond routine clinical measures remain uncertain.

PD progression varies substantially between individuals, and longitudinal clinical and molecular information may help characterize this heterogeneity (9–11). However, an association with clinical severity does not establish incremental prediction of future worsening.

We examined contemporaneous cytokine-score associations in PD and compared cytokine-only, clinical-only and combined models for prediction of next-visit motor-score worsening. Because of the modest sample size and limited validation data, the analyses were considered exploratory. The measured analytes are described throughout as cytokines in plasma EV-enriched preparations, without assuming an exclusively vesicular source.

## Materials and methods

### Study cohort and clinical assessments

This prospective longitudinal study enrolled 62 participants with PD and 21 healthy controls at a single center. PD was diagnosed according to the UK Parkinson’s Disease Society Brain Bank criteria (12). The cohort eligibility criteria specified Hoehn and Yahr stages I–III. Participants with atypical parkinsonism or comorbidities likely to affect motor or cognitive assessments were excluded. Controls had no major neurodegenerative disorder or functional disability. Participants were generally followed annually for clinical evaluations and blood sampling; those with PD who wished to continue were invited for extended follow-up. Controls contributed baseline descriptive information and the exploratory cytokine group-by-visit comparison; clinical-outcome association and prediction analyses were restricted to PD participants.

Motor function was assessed using the original Unified Parkinson’s Disease Rating Scale, with motor examination scores reported consistently as UPDRS Part III (UPDRS-III). Motor examinations were performed during routine outpatient follow-up on usual treatment, with ON-medication status confirmed by patient report. OFF assessments were not deliberately induced because of concerns about mobility and fall safety. The exact interval since the most recent antiparkinsonian dose was not recorded. Available additional measures included UPDRS Part II, tremor, akinetic-rigid and postural instability/gait difficulty (PIGD) scores, the Mini-Mental State Examination (MMSE), and the Montreal Cognitive Assessment (MoCA).

Body mass index (BMI), education, diabetes, smoking, inflammatory or infectious disease history, levodopa equivalent daily dose (LEDD), and recorded systemic steroid/nonsteroidal anti-inflammatory drug (NSAID) exposure were added to the clinical dataset. **Table 1** distinguishes never, former and current smokers; former and current smoking were combined as ever-smoking for modeling. Disease duration was approximated as the calendar year of the first recorded assessment minus the recorded onset year. Visit-specific medication records were aligned by nominal visit order, with prescription proxies used for some unavailable entries. Baseline BMI, education, smoking and disease-history data were abstracted for the baseline or sampling period.

**Table 1:** Baseline demographic and clinical characteristics.

Characteristic	HC (n = 21)	PD (n = 62)
Age, years	62.43 ± 6.70	66.15 ± 6.50
Male sex, n (%)	10 (47.6)	33 (53.2)
BMI, kg/m²	25.43 ± 4.72	23.86 ± 3.41
Education, years	11.43 ± 3.68	10.63 ± 4.29
Diabetes mellitus, n (%)	11 (52.4)	16 (26.2)^a^
Smoking status, n (%)		
Never	8 (38.1)	39 (62.9)
Former	2 (9.5)	22 (35.5)
Current	11 (52.4)	1 (1.6)
Baseline MMSE score	28.19 ± 1.75	27.79 ± 1.87
Disease duration at baseline, years	—	1.97 ± 1.63^b^
Baseline MoCA score	Not available	23.47 ± 4.06^c^
Baseline UPDRS Part III score	—	19.48 ± 9.24
LEDD at first assessment, mg/day	—	370.62 ± 211.10^a^
UPDRS-based stage groups, n (%)	—	
1	—	3 (4.8)
1.5	—	4 (6.5)
2	—	25 (40.3)
2.5	—	24 (38.7)
3	—	6 (9.7)

Values are mean ± standard deviation or n (%). Percentages use the number of participants with available data for each variable.

^a^Data were available for 61 participants with PD for diabetes mellitus and first-assessment LEDD; disease duration was available for 60 participants, MoCA was available for 49 participants, and all remaining reported variables were complete.

^b^Disease duration was approximated as the calendar year of the first recorded assessment minus the recorded year of PD onset. Data were available for 60 participants; two uncertain or missing onset years were excluded. The first recorded assessment was treated as the baseline visit under the study’s ordinal visit-alignment assumption.

^c^Baseline MoCA was available for 49 participants with PD. A corresponding HC summary was unavailable.

BMI, body mass index; HC, healthy controls; LEDD, levodopa equivalent daily dose; MMSE, Mini-Mental State Examination; MoCA, Montreal Cognitive Assessment; PD, Parkinson’s disease; UPDRS, Unified Parkinson’s Disease Rating Scale (original version). —, not applicable.

The study was approved by the Joint Institutional Review Board of Taipei Medical University (N202205008). Written informed consent was obtained from all participants.

### Blood collection and cytokine measurement

Blood was collected during routine outpatient follow-up without a fasting requirement or a standardized time of day. Plasma was obtained by centrifugation at 3,000 × g for 15 min at 4 °C and stored at −80 °C before EV isolation. ExoQuick polymer precipitation (System Biosciences) was used to obtain EV-enriched preparations. Particle measurements, transmission electron microscopy and Western blotting supported the presence of EV-containing material (**Supplementary Figure S1**), but did not establish purified exosomes or distinguish intraluminal, surface-associated and co-isolated non-vesicular cytokines (6, 13).

Cytokines were measured by BIOTOOLS using a 15-plex chemiluminescent immunoassay (MEK1007; Boster Biological Technology). The panel comprised IL-1α, IL-1β, IL-2, IL-4, IL-5, IL-6, IL-10, IL-12, IL-13, IL-15, IL-17, IL-23, IFNγ, TNFα and TNFβ. Detailed preparation and assay procedures are provided in the **Supplementary Material**.

The multiplex assay has lower limits of detection and quantification (LOD/LLOQ). For GEE, the corresponding records were excluded only from models requiring that cytokine, without substituting zero or a fraction of an assay limit. For ML, these values were treated as unavailable: only earlier within-participant cytokine values could be carried forward, and remaining missing predictors were median-imputed within the training folds with missingness indicators. Additional details are provided in the **Supplementary Material**.

### Outcomes and statistical analysis

The primary prediction endpoint was an increase of at least three points in original UPDRS-III between consecutive follow-up visits. A threshold of at least five points was evaluated in a sensitivity analysis. These operational definitions were informed by published clinically important difference estimates (14, 15), but were not interpreted as medication-independent individual neurodegenerative progression. Participants were generally followed annually.

For the exploratory comparison between PD and controls, positive cytokine concentrations at Visits 1–3 were log_2_-transformed. Descriptive group differences were displayed after standardizing each analyte using one pooled mean and sample standard deviation across both groups and all three visits. Separate Gaussian GEE models for each cytokine included group, categorical visit, group-by-visit interactions, age and sex, with participant clustering, an exchangeable working correlation and robust standard errors. Benjamini–Hochberg correction was applied across the 30 visit-specific interaction contrasts; the 15 joint group-by-visit tests formed a separate correction family.

Descriptive PD trajectories at Visits 1–5 used clinical scores and log_2_-transformed cytokine concentrations standardized against each measure’s fixed mean and sample standard deviation across all available observations over those visits. Visit means used available observations without imputation or restriction to records complete for model covariates. These plots were descriptive; the corresponding means, sample sizes and standardization values are provided in Supplementary_Statistical_Tables.xlsx.

For the supporting longitudinal analysis, separate Gaussian GEE models with an identity link examined each of 15 log_2_-transformed cytokines and seven clinical outcomes at Visits 1–5. Models included categorical visit, cytokine concentration and their interaction, adjusted for age, sex, BMI, education, diabetes, ever-smoking, inflammatory or infectious disease history, baseline disease duration, and visit-aligned LEDD. Participant clusters, an exchangeable working correlation and robust sandwich standard errors accounted for repeated observations. Each model used records complete for its required variables without imputation. Baseline disease duration was entered as a continuous participant-level covariate; records without usable duration were excluded. Four interaction coefficients were jointly tested for each cytokine–outcome pair; Benjamini–Hochberg FDR correction was applied across all 105 omnibus tests. Full constituent estimates, pointwise confidence intervals, raw and adjusted P values, and sensitivity analyses are reported in the **Supplementary Material** and the accompanying workbook Supplementary_Statistical_Tables.xlsx. These tests assess differences in contemporaneous associations across visits, rather than subsequent clinical change.

### Machine learning and incremental prediction

The prediction dataset comprised 147 intervals from 62 participants, covering Visits 1→2, 2→3 and 3→4 with observed current and next-visit UPDRS-III scores. Clinical-only, cytokine-only and combined predictor sets were evaluated on the same eligible intervals and outer partitions. Clinical and combined models shared the same clinical domains: age, sex, approximate disease duration, motor and cognitive histories, LEDD, BMI, education, diabetes, ever-smoking, inflammatory or infectious history, and recorded steroid/NSAID exposure. The cytokine models used all 15 analytes and their temporal representations. Predictor sets contained 68 clinical, 105 cytokine and 173 combined derived columns before imputation indicators; these counts do not represent independent clinical variables. Feature definitions and the availability limitations of medication and history records are specified in the **Supplementary Material**.

Positive cytokine concentrations were log_2_-transformed. Past-only within-participant carry-forward preceded temporal feature construction; remaining missing predictors were handled by training-fold median imputation, missingness indicators and standardization. Values below assay limits followed the handling described above.

Five outer and three inner participant-grouped cross-validation folds were used. The same partitions were reused across predictor sets and both outcome thresholds. All intervals from a participant remained together. Imputation, scaling, tuning and sigmoid probability calibration were fitted within the relevant training data. The main presentation uses radial basis function support vector machines (RBF-SVM), independently tuned for each predictor set over C = 0.1, 1 and 10 and gamma = scale, 0.01 and 0.1. This choice followed exploratory algorithm comparisons and was not prespecified before those comparisons. Fixed RBF-SVM, logistic regression, linear SVM, random forest and histogram gradient boosting were retained in the supplementary benchmark.

The principal discrimination measure was the AUC from pooled calibrated out-of-fold probabilities. Combined-minus-clinical differences were calculated from paired predictions. Confidence intervals used 2,000 participant-level bootstrap resamples of the fixed out-of-fold predictions, retaining participants’ repeated intervals. These intervals condition on the fitted predictions and splits, do not incorporate model refitting or selection uncertainty, and were not adjusted for the exploratory model comparisons. The revised models have not undergone a new temporal or external validation. Reporting was organized with reference to TRIPOD+AI (16).

## Results

### Cohort characteristics

Baseline characteristics are shown in **Table 1**. Participants with PD had a mean age of 66.15 ± 6.50 years and an approximate disease duration of 1.97 ± 1.63 years among 60 participants with usable onset data. Baseline UPDRS-III was 19.48 ± 9.24. First-assessment LEDD was 370.62 ± 211.10 mg/day, and MoCA was 23.47 ± 4.06. The prediction dataset contained 61 worsening intervals at the three-point threshold and 48 at the five-point threshold, among the same 147 intervals.

### Cytokine profiles in participants with PD and controls

To place the PD cytokine profiles in context, we compared participants with PD and healthy controls over the first three visits. Mean log_2_ cytokine profiles differed in direction across analytes: nine cytokines had lower levels in PD and six had higher levels at each visit (**Figure 1A**). IL-6, IL-13 and IFN-γ showed particularly pronounced negative standardized group differences. Age- and sex-adjusted GEE models then examined whether changes from Visit 1 differed between groups. One of the 30 visit-specific interaction contrasts had a nominal P < 0.05, but none met the FDR threshold (**Figures 1B, C**); none of the 15 joint interaction tests met its separate FDR threshold.

**Figure 1:**
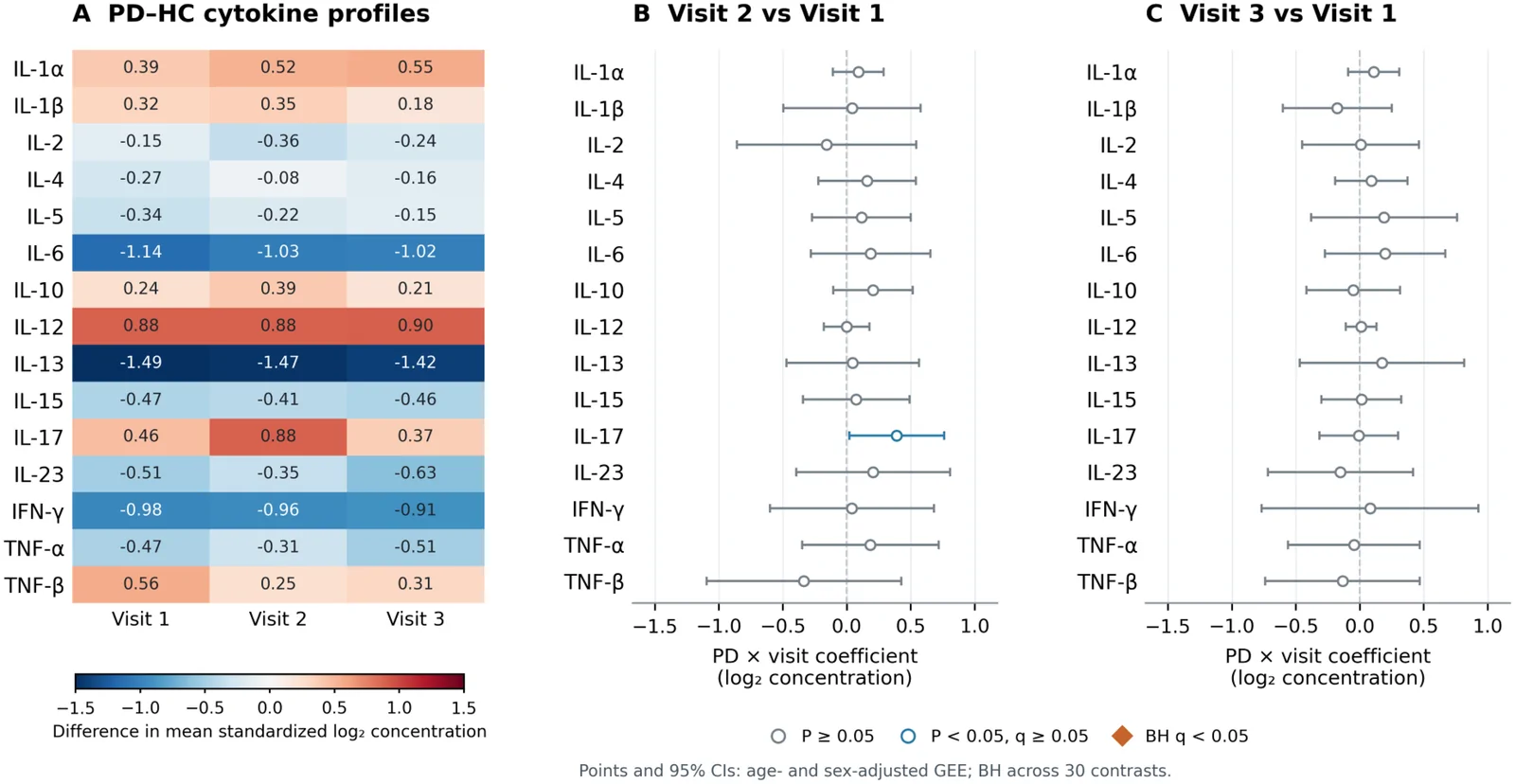
Cytokine profiles in participants with PD and healthy controls. **(A)** Differences in mean standardized log_2_ cytokine concentrations between PD and HC at Visits 1–3. Standardization used each analyte’s fixed mean and sample SD across both groups and all three visits; positive values indicate higher mean levels in PD. **(B, C)** PD-by-visit coefficients for Visit 2 and Visit 3 relative to Visit 1, from separate age- and sex-adjusted Gaussian GEE models with participant clustering, exchangeable correlation and robust covariance. Points and horizontal bars show estimates and pointwise 95% confidence intervals. Gray circles indicate raw P ≥ 0.05, blue circles raw P < 0.05 with BH-adjusted q ≥ 0.05, and orange diamonds BH-adjusted q < 0.05 across all 30 contrasts. No contrast met the FDR threshold. All panels use available measurements without imputation. PD, Parkinson’s disease; HC, healthy controls.

### Longitudinal clinical and cytokine profiles

To characterize the clinical and cytokine profiles underlying the association and prediction analyses, we summarized available PD measurements across Visits 1–5 using a fixed standardization for each measure. Mean UPDRS-III, akinetic-rigid and PIGD scores were higher at later visits, whereas tremor, UPDRS-II and cognitive scores varied across follow-up (**Figure 2A**). Most cytokines also showed higher mean log_2_ concentrations at later visits, with differences in the magnitude and pattern of change between analytes (**Figure 2B**).

**Figure 2:**
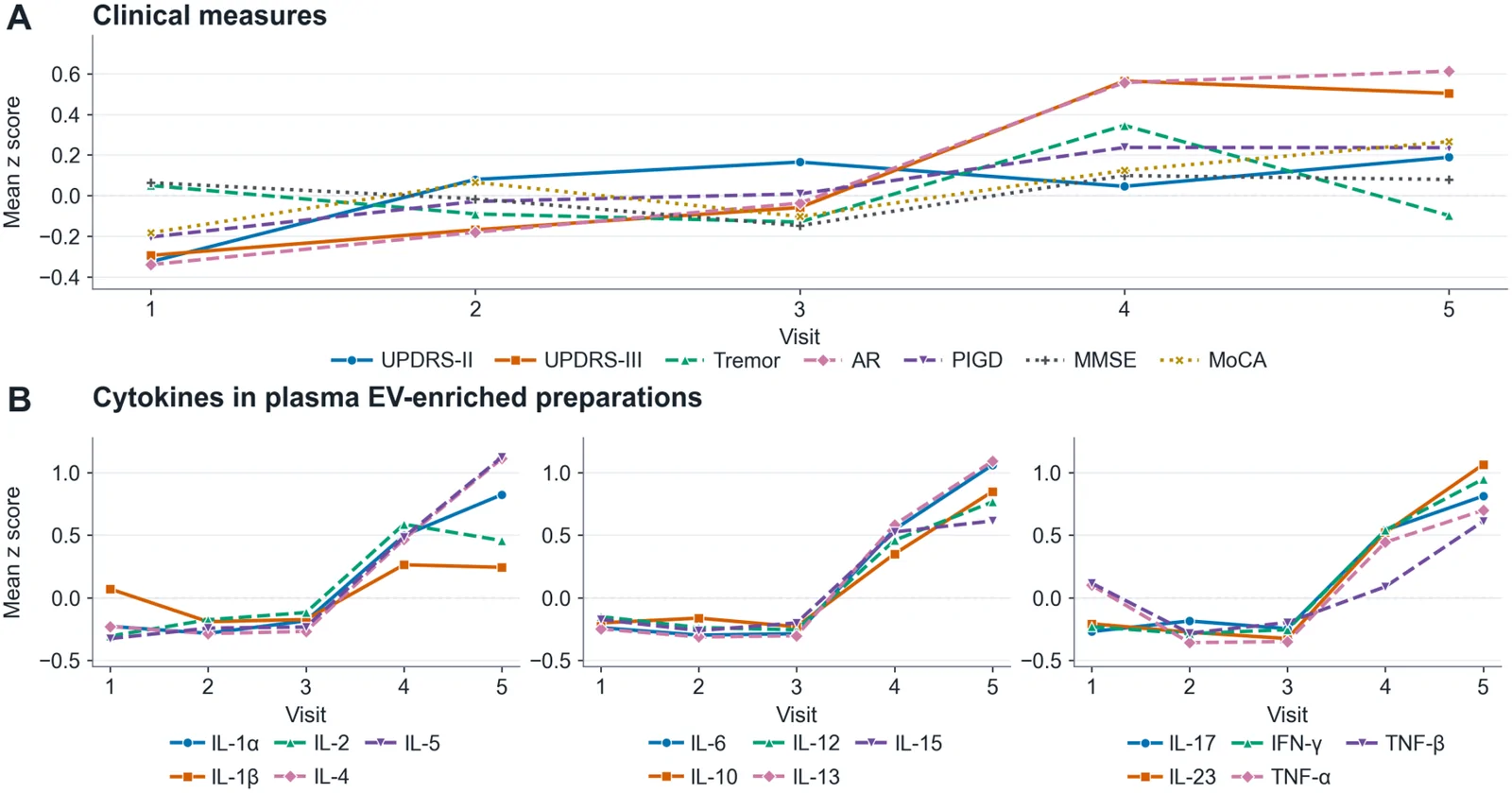
Longitudinal clinical and cytokine trajectories in PD. **(A)** Mean standardized clinical scores at Visits 1–5. **(B)** Mean standardized log_2_ cytokine concentrations in plasma EV-enriched preparations, displayed in three groups for readability. For each measure, one fixed mean and sample SD from all available PD observations across Visits 1–5 were used for standardization. Higher MMSE and MoCA values indicate better cognitive performance; higher motor scores indicate greater impairment. Each visit mean uses the available observations for that measure, without imputation. Sample sizes and numerical summaries are provided in workbook worksheet Figure2_Trajectories. AR, akinetic-rigid score; PIGD, postural instability and gait difficulty; MMSE, Mini-Mental State Examination; MoCA, Montreal Cognitive Assessment; UPDRS, original Unified Parkinson’s Disease Rating Scale.

### Contemporaneous cytokine associations

The 105 duration-adjusted GEE models included 57–59 participants and 179–220 observations, depending on the cytokine–outcome pair. Of the 105 omnibus interactions, 28 had raw P < 0.05 and 8 met the FDR threshold: IL-17 with UPDRS-III; IL-12 with tremor; IL-17 with akinetic-rigid; IL-23 with akinetic-rigid; IL-4 with PIGD; IL-12 with PIGD; IL-1β with MoCA; IL-15 with MoCA. Adjusted P values for these pairs ranged from 1.885e-05 to 0.03941. These joint tests identify differences in cytokine–score slopes across visits, not uniformly positive or negative associations. Effect magnitudes, nominal significance and FDR findings are shown in **Figure 3**, with all 105 omnibus results and all 420 constituent interaction contrasts in the **Supplementary Tables**. None of the 525 total visit-specific slopes met their separate FDR threshold. Results were sensitive to the cytokine concentration scale.

**Figure 3:**
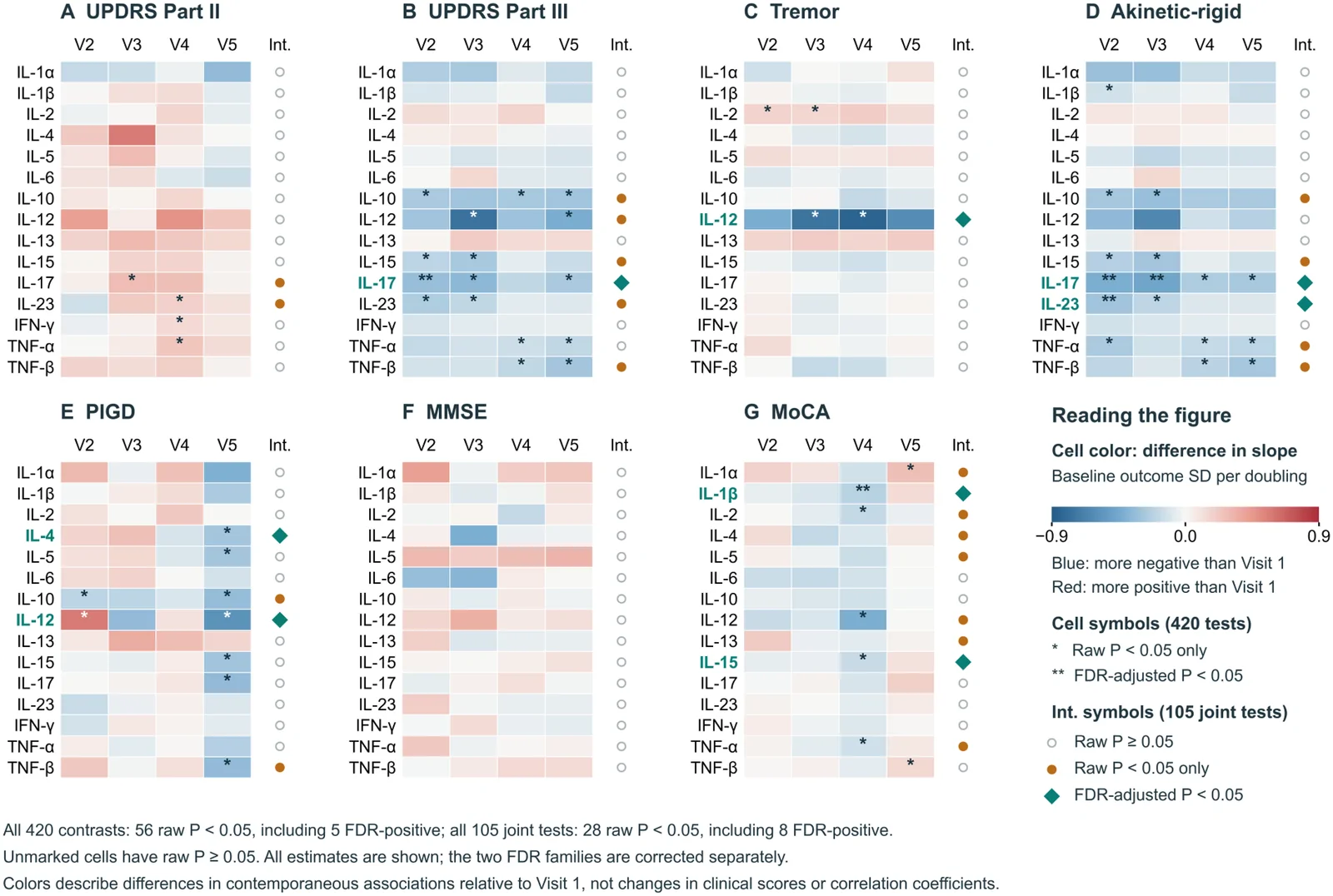
Magnitude and direction of cytokine by visit interactions. Panels show **(A)** UPDRS Part II, **(B)** UPDRS Part III, **(C)** tremor, **(D)** akinetic-rigid score, **(E)** postural instability/gait difficulty (PIGD), **(F)** Mini-Mental State Examination (MMSE), and **(G)** Montreal Cognitive Assessment (MoCA). All 420 interaction coefficients compare Visits 2–5 with Visit 1. Colors encode differences in cytokine–score slopes divided by the fixed baseline outcome SD, per doubling of cytokine concentration; blue indicates a more negative slope and red a more positive slope than at Visit 1. One asterisk denotes raw P < 0.05 only; two asterisks denote BH-adjusted P < 0.05 across 420 contrasts. Of 420 contrasts, 56 had raw P < 0.05, including 5 meeting that FDR threshold. The separate Int. columns show all 105 omnibus tests: gray circles indicate raw P ≥ 0.05, amber circles raw P < 0.05 only, and teal diamonds the 8 pairs with BH-adjusted P < 0.05 across 105 tests. There were 28 nominally significant omnibus tests. Colors represent standardized interaction effects, not correlation coefficients or annual score changes. Models adjusted for age, sex, BMI, education, diabetes, ever-smoking, inflammatory/infectious history, baseline disease duration and visit-aligned LEDD. Full estimates and pointwise confidence intervals are in workbook **Tables S3B** and **S3C**.

### Primary prediction analysis

To assess whether longitudinal cytokine measurements could complement clinical information in predicting motor worsening, we compared clinical-only, cytokine-only and combined RBF-SVM models using the same 147 follow-up intervals from 62 participants. The outcome was an increase of at least three points in UPDRS-III at the next visit. Models used current and previous measurements and were tuned and evaluated using participant-grouped nested cross-validation, with all intervals from a participant kept together. Clinical-only, cytokine-only and combined models yielded pooled out-of-fold AUCs of 0.733 (95% CI 0.644 to 0.812), 0.722 (0.628 to 0.806) and 0.784 (0.701 to 0.857), respectively (**Table 2**; **Figure 4A**). Among the three RBF-SVM predictor sets, the combined model had the highest observed AUC for distinguishing intervals with and without subsequent motor worsening. Adding cytokine measurements to clinical history was associated with a numerical increase in AUC of 0.051 (95% CI −0.009 to 0.112) in the paired combined-minus-clinical comparison.

**Table 2:** Discrimination of the tuned RBF SVM models.

Predictor set	≥3 points AUC or ΔAUC (95% CI)	≥5 points AUC or ΔAUC (95% CI)
Clinical only	0.733 (0.644 to 0.812)	0.743 (0.648 to 0.831)
Cytokine only	0.722 (0.628 to 0.806)	0.675 (0.563 to 0.772)
Combined	0.784 (0.701 to 0.857)	0.790 (0.701 to 0.870)
Combined minus clinical	0.051 (−0.009 to 0.112)	0.047 (−0.023 to 0.112)

The same 62 participants contributed 147 intervals to each endpoint; there were 61 events at ≥3 points and 48 at ≥5 points. All AUCs use pooled calibrated out-of-fold probabilities from five outer participant-grouped folds. Confidence intervals use 2,000 participant-level bootstrap resamples of fixed predictions and do not incorporate refitting, split or model-selection uncertainty. Paired ΔAUC compares combined and clinical-only predictions within each endpoint. Both endpoints use the median-imputation pipeline. AUC, area under the receiver operating characteristic curve; CI, confidence interval; RBF, radial basis function; SVM, support vector machine.

**Figure 4:**
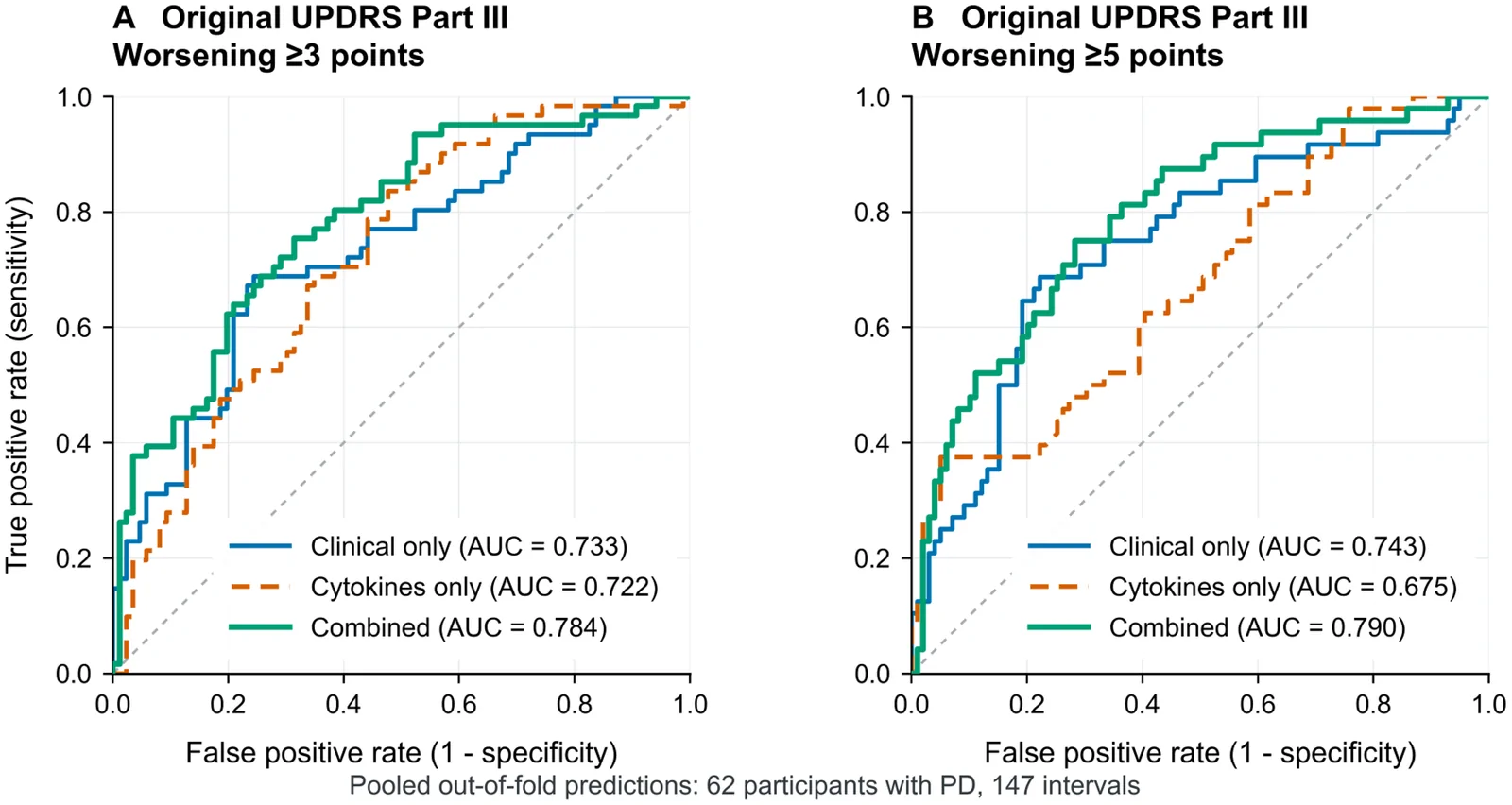
Prediction of next visit motor score worsening. Receiver operating characteristic curves for clinical-only, cytokine-only and combined tuned RBF-SVM models. Panel **(A)** uses worsening of at least three points in original UPDRS Part III; panel **(B)** uses at least five points. Curves are calculated from pooled calibrated out-of-fold probabilities from participant-grouped nested cross-validation and use the same 147 intervals from 62 participants. The dashed diagonal indicates chance discrimination. AUCs and conditional participant-bootstrap confidence intervals are reported in **Table 2**. The five-point analysis is a sensitivity analysis within the same cohort, not an independent validation.

### Sensitivity to the worsening threshold

To evaluate whether the prediction findings extended to larger changes in motor impairment, we repeated the analysis using a more stringent definition of worsening, an increase of at least five points in UPDRS-III. The same 147 intervals and participant-grouped cross-validation folds were retained, and models were retuned for the revised outcome. Forty-eight intervals met this definition, compared with 61 at the primary three-point threshold. Clinical-only, cytokine-only and combined models achieved pooled out-of-fold AUCs of 0.743 (95% CI 0.648 to 0.831), 0.675 (0.563 to 0.772) and 0.790 (0.701 to 0.870), respectively (**Table 2**; **Figure 4B**). The combined model again had the highest AUC among the three RBF-SVM predictor sets, with a paired combined-minus-clinical difference of 0.047 (95% CI −0.023 to 0.112). Thus, the numerical advantage of combining cytokine and clinical information was also observed when worsening required a larger increase in motor scores.

Results for the reported individual-family models are provided in the **Supplementary Material**.

## Discussion

This study examined cytokines in plasma EV-enriched preparations alongside longitudinal clinical assessments in PD. Eight cytokine-by-visit interactions passed correction across the 105 primary GEE tests after adjustment including disease duration. In the updated prediction analysis, combining cytokine and clinical features produced RBF-SVM AUCs of 0.784 and 0.790 at the three- and five-point worsening thresholds. The corresponding clinical-only AUCs were 0.733 and 0.743. Comparison with an optimized clinical baseline and reporting of both thresholds make the predictive contribution of the cytokine measurements more directly assessable.

The GEE and prediction analyses answer different questions. GEE identified variation across visits in contemporaneous associations, involving cytokines with motor domains and cognition. It did not establish temporal precedence or isolate within-person effects. The prediction models used current and past information to estimate next-visit motor-score worsening. A cytokine can contribute to a multivariable prediction model without a significant individual GEE term, and a significant GEE interaction does not establish incremental prediction. The dependence of the GEE findings on concentration scale and the absence of FDR-significant total slopes further support an exploratory interpretation.

The matched clinical-only comparison addresses the possibility that motor-score history and clinical severity account for most discrimination. RBF-SVM was emphasized after exploratory comparisons for continuity with the original SVM framework and comparison of predictor sets within a common algorithm. The supplementary benchmark is reported to make this choice transparent. Independent data are needed to evaluate whether the observed numerical gain persists beyond model development.

Cytokines were not measured in matched unfractionated plasma. We therefore cannot determine whether conventional plasma assays would show similar associations or whether EV enrichment adds value. Longitudinal blood-cytokine evidence is mixed: plasma IL-6, IL-17A and TNF-α were not independently associated with motor progression in adjusted analyses in one cohort (17), whereas serum immune profiles were associated with subsequent motor trajectories in another (18). These studies provide context but do not substitute for a direct comparison within the present cohort. Future studies should compare matched unfractionated plasma and EV-enriched preparations using the same analytical and prediction-validation framework. The findings do not demonstrate EV-specific cytokine localization or superiority over conventional plasma testing.

ON-medication status during the motor examinations was confirmed by patient report. Nevertheless, exact dose-to-examination intervals were not recorded, and treatment changes or variation in response can influence between-visit motor scores. LEDD was included in both the clinical and combined prediction models and in the GEE adjustment set, but cannot reconstruct acute dose timing or eliminate medication-related effects on scores or cytokines. The five-point sensitivity analysis evaluates dependence on the outcome threshold; it does not validate medication-independent progression. Gait and balance components differ in dopaminergic responsiveness (19), and medication effects on cognition vary by task (20). PIGD and cognitive outcomes therefore cannot be treated as medication-independent controls.

Other limitations include the modest single-center cohort, high-dimensional temporal predictors, incomplete follow-up and sparse later GEE visits. Baseline disease duration was included in both GEE and the clinical prediction models, but its calendar-year approximation does not capture exact time since onset or individual disease stage. Some clinical fields and prescription proxies were assembled retrospectively, so strictly prospective availability at every prediction origin was not verified. The primary bootstrap intervals omit refitting and exploratory selection uncertainty. Calibration and clinical utility require further assessment, and the updated models have no new temporal or external validation.

Polymer precipitation can co-isolate soluble proteins, lipoproteins, protein aggregates, immune complexes and other non-vesicular particles, and the characterization did not locate cytokines within or on EVs. Sampling followed outpatient schedules without a fasting requirement, a standardized time of day, or standardized meal and dose intervals. Although plasma was stored at −80 °C, samples collected in earlier years had longer storage durations before analysis. Storage duration was not sufficiently characterized for adjustment, and its potential influence on measured cytokine concentrations cannot be excluded. The severity and activity of recorded inflammatory or infectious conditions were unavailable, preventing assessment of their intensity or active status at each blood draw. The assay lower limits mean that some low cytokine concentrations could not be quantified. Excluding these left-censored values from GEE, or treating them as unavailable predictors in ML, may alter estimated associations and predictive performance, particularly for cytokines with more below-limit observations. The lack of complete applicable early-visit LOD/LLOQ information prevented a censoring-specific sensitivity analysis. Residual confounding, informative missingness and bias related to assay limits remain possible.

## Conclusion

Longitudinal cytokine measurements in plasma EV-enriched preparations showed 8 FDR-significant cytokine-by-visit interactions after adjustment including baseline disease duration. Combined RBF-SVM models achieved AUCs of 0.784 and 0.790 for next-visit UPDRS-III worsening at the three- and five-point thresholds. These findings support further evaluation of cytokine measurements alongside clinical histories, with independent validation and matched plasma comparisons needed before clinical use.

## Data Availability

Access to participant-level data is subject to approval by the Joint Institutional Review Board of Taipei Medical University. Analysis code and trained model artifacts are available from the corresponding author upon reasonable request. Aggregate GEE and ML results are provided in the accompanying workbook **Supplementary File 2**.

## Supplementary material

Supplementary File 1 Assay procedures, complete GEE and machine-learning methods, supporting numerical summaries, and **Supplementary Figure S1**.

Supplementary File 2 Complete aggregate results for **Tables S3A–S3I** and **S4A–S4C**, together with data summaries supporting main **Figures 1** and **2**.

## References

[1] Tansey MG Wallings RL Houser MC Herrick MK Keating CE Joers V . Inflammation and immune dysfunction in Parkinson disease. Nat Rev Immunol. (2022) 22:657–73. doi: 10.1038/s41577-022-00684-6 35246670 PMC8895080

[2] Hong CT Chung CC Hsieh YC Chan L . Plasma extracellular vesicle pathognomonic proteins as the biomarkers of the progression of Parkinson's disease. Biosci Trends. (2025) 19:116–24. doi: 10.5582/bst.2024.01369 39924179

[3] Jiang C Hopfner F Katsikoudi A Hein R Catli C Evetts S . Serum neuronal exosomes predict and differentiate Parkinson's disease from atypical parkinsonism. J Neurology Neurosurgery Psychiatry. (2020) 91:720–9. doi: 10.1136/jnnp-2019-322588 32273329 PMC7361010

[4] Kluge A Bunk J Schaeffer E Drobny A Xiang W Knacke H . Detection of neuron-derived pathological α-synuclein in blood. Brain. (2022) 145:3058–71. doi: 10.1093/brain/awac115 35722765

[5] Fitzgerald W Freeman ML Lederman MM Vasilieva E Romero R Margolis L . A system of cytokines encapsulated in extracellular vesicles. Sci Rep. (2018) 8:8973. doi: 10.1038/s41598-018-27190-x 29895824 PMC5997670

[6] Welsh JA Goberdhan DCI O'Driscoll L Buzas EI Blenkiron C Bussolati B . Minimal information for studies of extracellular vesicles (MISEV2023): From basic to advanced approaches. J Extracell Vesicles. (2024) 13:e12404. doi: 10.1002/jev2.12404 38326288 PMC10850029

[7] Chan L Chung CC Chen JH Yu RC Hong CT . Cytokine profile in plasma extracellular vesicles of Parkinson's disease and the association with cognitive function. Cells. (2021) 10:604. doi: 10.3390/cells10030604 33803292 PMC7999703

[8] Chan L Chung CC Yu RC Hong CT . Cytokine profiles of plasma extracellular vesicles as progression biomarkers in Parkinson's disease. Aging (Albany NY). (2023) 15:1603–14. doi: 10.18632/aging.204575 36897204 PMC10042681

[9] Severson KA Chahine LM Smolensky LA Dhuliawala M Frasier M Ng K . Discovery of Parkinson's disease states and disease progression modelling: a longitudinal data study using machine learning. Lancet Digit Health. (2021) 3:e555–64. doi: 10.1016/s2589-7500(21)00101-1 34334334

[10] Fereshtehnejad S-M Zeighami Y Dagher A Postuma RB . Clinical criteria for subtyping Parkinson’s disease: biomarkers and longitudinal progression. Brain. (2017) 140:1959–76. doi: 10.1093/brain/awx118 28549077

[11] Simuni T Siderowf A Lasch S Coffey CS Caspell-Garcia C Jennings D . Longitudinal change of clinical and biological measures in early Parkinson's disease: Parkinson's Progression Markers Initiative Cohort. Mov Disord. (2018) 33:771–82. doi: 10.1002/mds.27361 29572948 PMC6001458

[12] Hughes AJ Daniel SE Kilford L Lees AJ . Accuracy of clinical diagnosis of idiopathic Parkinson's disease: a clinico-pathological study of 100 cases. J Neurol Neurosurg Psychiatry. (1992) 55:181–4. doi: 10.1136/jnnp.55.3.181 1564476 PMC1014720

[13] Théry C Witwer KW Aikawa E Alcaraz MJ Anderson JD Andriantsitohaina R . Minimal information for studies of extracellular vesicles 2018 (MISEV2018): a position statement of the International Society for Extracellular Vesicles and update of the MISEV2014 guidelines. J Extracell Vesicles. (2018) 7:1535750. doi: 10.1080/20013078.2018.1535750 30637094 PMC6322352

[14] Shulman LM Gruber-Baldini AL Anderson KE Fishman PS Reich SG Weiner WJ . The clinically important difference on the unified Parkinson's disease rating scale. Arch Neurol. (2010) 67:64–70. doi: 10.1001/archneurol.2009.295 20065131

[15] Sánchez-Ferro Á Matarazzo M Martínez-Martín P Martínez-Ávila JC Gómez de la Cámara A Giancardo L . Minimal clinically important difference for UPDRS-III in daily practice. Mov Disord Clin Pract. (2018) 5:448–50. doi: 10.1002/mdc3.12632 30838303 PMC6336372

[16] Collins GS Moons KGM Dhiman P Riley RD Beam AL Van Calster B . TRIPOD+AI statement: updated guidance for reporting clinical prediction models that use regression or machine learning methods. BMJ. (2024) 385:e078378. doi: 10.1136/bmj-2023-078378 38626948 PMC11019967

[17] Chen SJ Chi YC Ho CH Yang WS Lin CH . Plasma lipopolysaccharide-binding protein reflects risk and progression of Parkinson's disease. J Parkinsons Dis. (2021) 11:1129–39. doi: 10.3233/JPD-212574 33720853

[18] Williams-Gray CH Wijeyekoon R Yarnall AJ Lawson RA Breen DP Evans JR . Serum immune markers and disease progression in an incident Parkinson's disease cohort (ICICLE-PD). Mov Disord. (2016) 31:995–1003. doi: 10.1002/mds.26563 26999434 PMC4957620

[19] Curtze C Nutt JG Carlson-Kuhta P Mancini M Horak FB . Levodopa is a double-edged sword for balance and gait in people with Parkinson's disease. Mov Disord. (2015) 30:1361–70. doi: 10.1002/mds.26269 26095928 PMC4755510

[20] Cools R Barker RA Sahakian BJ Robbins TW . Enhanced or impaired cognitive function in Parkinson's disease as a function of dopaminergic medication and task demands. Cereb Cortex. (2001) 11:1136–43. doi: 10.1093/cercor/11.12.1136 11709484

