# Supplementary material for: Longitudinal cytokines in plasma extracellular vesicle enriched preparations and motor worsening in Parkinson disease
Source: Front Immunol. 2026 Oct 21;17:1973023. doi: 10.3389/fimmu.2026.1973023 (PMC13732178; doi:10.3389/fimmu.2026.1973023)
Supplement: Supplementary file 1 [file SupplementaryFile1.docx]

Supplementary Material

# Longitudinal cytokines in plasma extracellular vesicle enriched preparations and motor worsening in Parkinson disease

This supplement provides assay details, complete GEE and machine-learning methods, numerical summaries and supporting figures. The accompanying workbook Supplementary_Statistical_Tables.xlsx contains the complete numerical results for Tables S3A–S3I and S4A–S4C; the sections below describe the contents of those worksheets. The workbook is a separate supplementary file and accompanies this document. It does not contain participant-level data.

# Supplementary assay methods

## Plasma preparation and EV enrichment

Blood was collected during routine follow-up clinic visits without a fasting requirement or a standardized time of day. Plasma was obtained by centrifugation at 3,000 × g for 15 min at 4°C and stored at −80°C before EV isolation. Thrombin Plasma Prep reagent (TMEXO-1; System Biosciences) was used before ExoQuick precipitation (EXOQ5A-1; System Biosciences). ExoQuick reagent (63 µL per 250 µL plasma) was added, followed by incubation at 4°C for 30 min and centrifugation at 1,500 × g for 30 min. The recovered pellet was resuspended in 100 µL buffer.

Preparations were characterized using nanoparticle tracking analysis, transmission electron microscopy, and Western blotting for CD63, CD9, CD81, and TSG101. These measurements demonstrate EV-containing material, but do not establish a purified exosome population, exclude non-vesicular co-isolates, or establish the location of cytokines. Measurements are therefore described as cytokines in plasma EV-enriched preparations. MISEV2018 and MISEV2023 are cited in the main manuscript (references 6 and 13).

# Cytokine measurement and reporting

The panel measured IL-1α, IL-1β, IL-2, IL-4, IL-5, IL-6, IL-10, IL-12, IL-13, IL-15, IL-17, IL-23, IFN-γ, TNF-α, and TNF-β using the Q-Plex Human Cytokine HS Screen (15-Plex; MEK1007, BOSTER). Protein extracts were prepared from the EV-enriched material with RIPA at a 1:1 volume ratio. Samples were diluted 1:2 for the assay, and 50 µL of samples, calibration standards, and blank were loaded in duplicate. Seven-point calibration curves were used, and chemiluminescent signals were acquired with the Q-View Imager LS and analyzed with Q-View software. Each multiplex plate accommodated up to 40 specimens. Plasma samples from all PD study years were submitted together and assayed across multiple plates. Healthy-control samples were measured in two batches; controls were not included in the present clinical-outcome GEE or prediction models.

The study did not measure cytokines in matched unfractionated plasma or perform experiments distinguishing intraluminal, EV-surface-associated, and co-isolated non-vesicular cytokines. No inference of EV-specific cytokine localization or superiority over conventional plasma assays is made.

# Methods for main Figures 1 and 2

The exploratory PD–HC comparison used 62 participants with PD and 21 healthy controls at Visits 1–3. Each cytokine was summarized separately using finite positive concentrations; missing values, nonpositive values and unavailable-value codes were excluded without imputation. Cytokines were log₂-transformed. For Figure 1A, each analyte was standardized using a fixed mean and sample SD pooled across available measurements in both groups over Visits 1–3. Each heatmap cell is the PD mean z score minus the HC mean z score at the same visit, rather than a covariate-adjusted estimate.

For Figures 1B,C, separate Gaussian identity-link GEE models used log₂ cytokine concentration as the outcome, with PD status, categorical visit, PD-by-visit interactions, baseline age and sex as predictors. HC and Visit 1 were the reference categories. Participant clusters, an exchangeable working correlation and robust sandwich covariance accounted for repeated measurements. The interaction coefficients compare the between-group difference in change from Visit 1 to Visit 2 or 3, in log₂ concentration units. Pointwise 95% confidence intervals used normal approximations. All 30 interaction contrasts formed one Benjamini–Hochberg correction family, and the 15 two-degree-of-freedom joint interaction tests formed a separate family. All 15 models converged with full-rank designs. One visit-specific contrast had a nominal P < 0.05, and none passed its FDR threshold; no joint test passed its separate FDR threshold.

Figure 2 summarizes the PD cohort at Visits 1–5, independently of the PD–HC standardization. Each original clinical score and each log₂-transformed cytokine was standardized using its mean and sample SD across all available PD observations over these five visits. Visit-specific means used the available observations for that measure without imputation or a requirement for complete model covariates. Higher MMSE and MoCA scores indicate better cognitive performance, whereas higher motor scores indicate greater impairment. The trajectories are descriptive available-observation summaries; no trend significance tests were added for this figure.

The accompanying Supplementary_Statistical_Tables.xlsx provides the complete descriptive summaries, effective sample sizes, standardization values and group-comparison model results in worksheets Figure1_GroupMeans, Figure1_GEE and Figure2_Trajectories. Figure1_GEE distinguishes the 30-contrast FDR family from the 15-model joint-test family. Model-level joint results repeated alongside coefficient rows represent the same joint test and should not be counted more than once.

# Supplementary GEE methods

## GEE population and clinical measurements

The PD-only analysis included 62 participants and the first five nominal study visits, providing 310 candidate participant–visit records before model-specific exclusions. Healthy controls were not included in these clinical-outcome GEE analyses. The seven outcomes were original UPDRS Part II, original UPDRS Part III, tremor, akinetic-rigid and postural instability/gait difficulty (PIGD) scores, MMSE, and MoCA. Each of 15 cytokines was analyzed in a separate model for each outcome; the cytokines were not entered together in one model. The cytokine panel comprised IL-1α, IL-1β, IL-2, IL-4, IL-5, IL-6, IL-10, IL-12, IL-13, IL-15, IL-17, IL-23, IFNγ, TNFα, and TNFβ.

Visit was categorical, with Visit 1 as the reference. Cytokine and clinical measurements assigned the same visit number were treated as contemporaneous. The analysis did not include lagged cytokine exposures, clinical change scores, or person-mean decomposition. Accordingly, a cytokine-by-visit interaction represents a difference between visits in the population-average cytokine–score slope, rather than an annual clinical-score change or an isolated within-person effect. Participants were generally followed annually.

Original UPDRS Part III assessments were performed during routine outpatient follow-up on usual treatment, with ON-medication status confirmed by patient report. OFF assessments were not deliberately induced because of concerns about mobility and fall safety. The interval since the last antiparkinsonian dose was not recorded. ON-state assessment does not establish identical dose timing or treatment response across visits. PIGD and cognitive scores were not treated as medication-independent controls.

## Covariates and available-record analysis

The nine adjustment variables were age, sex, BMI, education, diabetes, ever-smoking, recorded inflammatory or infectious disease history, baseline disease duration, and visit-aligned LEDD. The first eight were fixed participant-level covariates; LEDD varied by nominal visit. Baseline disease duration was the calendar year of the first recorded assessment minus the recorded PD onset year, entered continuously in years and held constant across visits. Duration was available for 60 of the 62 participants; two missing or uncertain onset years remained missing. These participants were excluded through the model-specific complete-record rule, without duration imputation. Former and current smoking were combined for modeling because only one participant with PD was a current smoker; there were 23 ever-smokers and 39 never-smokers. Recorded inflammatory or infectious history was present in 8 of 62 participants (12.9%). The severity and activity of these conditions were unavailable, so this history variable did not establish active inflammation at each blood draw. Unknown values were retained as missing rather than automatically recoded as absence of disease or exposure.

The first supplied LEDD record was matched to Visit 1, and subsequent records were matched by visit order. One participant's first six LEDD entries were designated missing following investigator clarification; the first five therefore contributed no complete records to these GEE models. Daily LEDD does not identify exact dose-to-assessment or dose-to-draw intervals. Individual antiparkinsonian drug classes were not GEE covariates. Steroid and NSAID exposure was addressed by a sensitivity exclusion rather than added to the primary adjustment set.

Numeric missing-value codes, including 999, and nonnumeric unavailable values were treated as missing; documented zero values for covariates were retained. Records were included separately for each cytokine–outcome model when the outcome, cytokine and all nine covariates were available. Thus, observation counts differ among models, and completeness of the entire cytokine panel was not required. No imputation, carry-forward or concentration-based outlier exclusion was used in GEE. The missingness table reports counts that may overlap across variables.

The multiplex assay has analyte-specific lower detection and quantification limits. Sixty-four cytokine measurements originally coded as zero represented concentrations below detection or quantification limits and were treated as unavailable: 2 IFNγ, 4 IL-17, 30 IL-2 and 28 TNFβ measurements, all within Visits 1–5. These are 64 assay observations, not 64 participants. Values below a limit indicate left censoring rather than a true zero concentration. GEE excluded the corresponding records only for models requiring the affected cytokine; all other eligible observations were retained. No zero replacement, pseudocount, LOD/2 or LLOQ/2 substitution, imputation or carry-forward was applied in GEE. The applicable limits for all affected early-visit specimens could not be reconstructed from the available records, so no explicit left-censoring model was fitted. This handling is distinct from the training-fold missing-predictor procedure used in ML and is considered a limitation.

## Model fitting and effect units

Positive available cytokine concentrations were log₂-transformed without adding a pseudocount. Gaussian GEE models used an identity link, participant-level clusters, an exchangeable working correlation and robust sandwich covariance, implemented in statsmodels 0.14.6. For numerical conditioning, the exposure was centered and scaled during fitting, then estimates and covariance-derived uncertainty were converted back to clinical-score units per doubling of cytokine concentration. Age, BMI and education were centered and scaled in units of 10 years, 5 kg/m² and 5 years, respectively; LEDD was entered per 100 mg/day. Baseline disease duration was centered at 2 years and entered per 1 year. These linear rescalings did not change the substantive model.

Normal-approximation 95% confidence intervals were calculated for individual estimates, and joint hypotheses were assessed using chi-square Wald tests. The Gaussian mean model was also used for bounded cognitive scores and derived motor scores. All 525 primary and sensitivity fits converged and had full-rank designs and valid joint-test covariance matrices. Robust covariance accounts for within-participant correlation, but does not correct a misspecified mean model, unmeasured confounding, measurement error or informative dropout.

## Multiplicity and interpretation

The primary family comprised 105 four-degree-of-freedom omnibus tests of the four cytokine-by-visit interaction coefficients for Visits 2–5 versus Visit 1. Benjamini–Hochberg adjustment was applied across the complete 105-test family. This joint test asks whether the cytokine–score slope differs across visits; it has no single coefficient, direction or 95% confidence interval.

Three additional families were reported separately. First, a secondary five-degree-of-freedom global cytokine test jointly assessed the Visit 1 cytokine coefficient and all four interaction coefficients, with adjustment across 105 tests. Second, all 420 individual interaction contrasts were adjusted together. Third, the 525 total cytokine slopes at the five visits formed a descriptive adjustment family. A Visit 1 total slope is the model's main cytokine coefficient; at later visits it is the sum of that coefficient and the relevant interaction. Its standard error and confidence interval use the covariance between those coefficients.

Adjustment was performed separately for each family within each of the five model variants, yielding 20 defined families rather than one study-wide correction. Discoveries from these overlapping hypotheses were not added together. Confidence intervals are pointwise, not simultaneous or FDR-adjusted. Primary interpretation was based on the 105 omnibus interactions rather than selection from secondary tests or sensitivity variants. A negative interaction coefficient need not imply a negative total cytokine–score association. Higher MMSE and MoCA scores indicate better cognitive performance.

## Sensitivity and participant-influence analyses

Four sensitivity variants used (1) age- and sex-only adjustment on the same complete records as the fully adjusted model; (2) full adjustment with working independence; (3) full adjustment with untransformed cytokine concentrations and exchangeable correlation; and (4) full adjustment after excluding visits with documented systemic steroid or NSAID use. The exposure units in the raw-concentration variant are original concentration units, not cytokine doublings. The exclusion variant retained visits with unknown exposure documentation and cannot be interpreted as a verified medication-free sample.

For the 8 primary FDR-significant cytokine–outcome pairs, participants were also omitted one at a time. These 472 selected diagnostic fits assessed raw omnibus P values and changes in interaction coefficients relative to their full-model standard errors. FDR was not recalculated for these diagnostic fits. The refit specification was fixed before executing this revised analysis, but was not prospectively registered before the original study results were known.

# Supplementary GEE results

The 105 primary models adjusted for baseline disease duration in addition to the previous eight covariates. They included 57–59 participants and 179–220 observations, with 15–18 observations at Visit 5 in each model. All 525 primary and sensitivity models converged and had valid joint tests. Of the 105 primary omnibus interactions, 28 had raw P < 0.05 and 8 had BH-adjusted P < 0.05. Main Figure 3 displays the interaction estimates and significance patterns. Table S1 summarizes these pairs; full-precision results for all 105 pairs are in workbook Table S3A. Table S2 summarizes the sensitivity variants. Separately, the global cytokine family had 24 nominal and 13 FDR-significant tests, the 420 interaction contrasts had 56 nominal and 5 FDR-significant tests, and the 525 total slopes had 64 nominal and 0 FDR-significant tests.

The age/sex-only variant identified 14 FDR-significant omnibus interactions, retaining 8 of the 8 primary pairs. Working independence identified 7 FDR-significant pairs and retained 6 primary pairs. Exclusion of documented steroid/NSAID visits identified 7 FDR-significant pairs, retained 5 primary pairs and included 174–211 observations. Raw-concentration models identified 30 FDR-significant pairs and retained 6 primary pairs. Full values for all pairs in each variant are in workbook Table S3D; Table S3H summarizes the primary discoveries across variants. Dependence on exposure scale precludes describing the findings as uniformly robust.

Of 472 leave-one-participant-out diagnostic fits, 472 were valid and 472 retained raw omnibus P < 0.05. The maximum raw P was 0.0249971, and the largest interaction-coefficient change was 2.37697 full-model standard errors. The largest change occurred for PIGD–IL-4, indicating sensitivity of interaction magnitudes to participant omission. These selected, unadjusted diagnostics do not demonstrate FDR robustness or the absence of influential observations. Missing follow-up data, sparse later visits, below-assay-limit handling and incomplete control of treatment timing remain limitations.

## Additional clinical covariate documentation

Recorded inflammatory or infectious disease history was present in 8 of 62 participants (12.9%). Across the 310 candidate participant–visit records at Visits 1–5, documented systemic steroid use occurred in one participant at one visit, NSAID use in six participants at nine visits, and either exposure in seven participants at ten visits. These counts describe the GEE candidate records, not only the ML prediction-origin visits. Unknown medication documentation was retained as unknown. Baseline disease duration and MoCA availability are reported in main Table 1.

# Supplementary machine-learning methods

## Analysis population, intervals and outcomes

Only participants with Parkinson's disease were included in prediction modeling; healthy controls were not used as prediction samples. The dataset comprised 147 intervals from 62 participants. A participant could contribute more than one interval, so the interval count does not represent 147 independent individuals. The analysis retained the original development intervals from visits 1→2, 2→3 and 3→4. Participants were generally followed annually. Intervals were excluded when either the current or next UPDRS-III score was missing. No outcome was imputed.

For participant i at origin visit t, the binary outcome at threshold h was Y(i,t;h) = 1 if UPDRS-III(i,t+1) − UPDRS-III(i,t) ≥ h, and 0 otherwise. The main threshold was h = 3 (61 positive intervals); the sensitivity threshold was h = 5 (48 positive intervals). Improvement, no change and changes below the respective threshold were coded 0. Scores were from the original UPDRS Part III, not MDS-UPDRS. ON-medication status was confirmed by patient report during routine outpatient assessments; exact time since the preceding dose was unavailable. Raising the threshold assesses dependence on the outcome definition and does not remove medication or measurement variability.

## Predictor domains and longitudinal features

Three predictor sets were constructed on the same intervals: clinical-only, cytokine-only and their combined union. Cytokine measurements represented the polymer-precipitated plasma EV-enriched preparation; the model does not establish intravesicular localization or EV specificity. The 15 analytes were IL-1α, IL-1β, IL-2, IL-4, IL-5, IL-6, IL-10, IL-12, IL-13, IL-15, IL-17, IL-23, IFN-γ, TNF-α and TNF-β.

The seven clinical-score domains were UPDRS II, UPDRS III, tremor, akinetic-rigid and PIGD subscores, MMSE and MoCA. For a score or log2 cytokine measurement x(i,t), the following features were generated using the origin visit and earlier visits:

1. **Current measurement:** three visit-specific columns x_current,j for j = 1, 2, 3, where x_current,j = x(i,t) when t = j and is structurally missing otherwise.

2. **Previous measurement:** x_lag1 = x(i,t−1), missing at t = 1.

3. **Previous-visit change:** x_delta1 = x(i,t) − x(i,t−1), missing if t = 1 or either value is missing.

4. **Baseline measurement:** x_base = x(i,1).

5. **Change from baseline:** x_deltab = x(i,t) − x(i,1), missing if either value is missing.

This gives seven engineered columns per score or analyte: three current-visit columns plus four historical/change columns. For cytokines, differences are differences of log2 concentrations, corresponding to log2 concentration ratios when both values are observed or have been carried forward. Raw clinical-score histories were not globally forward-filled. Baseline and current features can be redundant at the first interval; the specified feature layout was retained rather than changed after reviewing performance. Features did not contain next-visit outcomes or cytokine measurements.

Age used the baseline age plus t−1 years, reflecting the nominal annual-visit assumption, with the same seven-column current/history/change representation. Sex was repeated in three visit-specific columns. Thus, the original clinical and demographic block contained 49 score columns, seven age columns and three sex columns. The following nine supplemented covariates were added, producing 68 clinical inputs:

- **LEDD:** origin-visit prescription-based dose, aligned by ordinal visit.

- **BMI and education years:** fixed baseline covariates.

- **Diabetes:** binary disease-history covariate.

- **Ever-smoking:** former or current smoking versus never smoking. Former and current categories were combined because only one participant was a current smoker; the model did not use three smoking categories.

- **Inflammatory/infectious disease history:** binary ever-history proxy, not confirmation of active inflammation at each blood draw.

- **Disease duration:** approximate baseline calendar-year difference between the first recorded assessment and recorded PD onset; the value was held constant across intervals. Duration was available for 60 participants; uncertain or missing onset years remained missing. This is not exact time since diagnosis or an annually updated disease-duration measure.

- **Systemic corticosteroid and NSAID exposure:** separate origin-visit prescription-exposure variables, aligned by ordinal visit. Prescription records do not verify ingestion or medication absence, and exposures were sparse.

The diabetes and inflammatory-history variables were treated as baseline proxies. Clinical-record ascertainment and timing considerations are described under Study cohort and clinical assessments in the main manuscript. Motor subscores represent relevant clinical domains; no separate categorical phenotype, Hoehn and Yahr variable or comprehensive non-motor-burden scale was included in the model. The cytokine block contained 15 × 7 = 105 inputs; the combined set contained 173. The complete predictor-column list is available with the analysis code from the corresponding author upon reasonable request.

## Missing values and scaling

Source values coded 999, non-numeric or non-finite entries, and unknown values were treated as missing. Missing covariates were not recoded to absence. Positive raw cytokine concentrations were log2-transformed, without applying a globally estimated z-score. Missing cytokine values could be filled from earlier visits of the same participant, with no backward filling from later cytokine measurements. The subsequent training-partition transformation appended one binary missingness indicator per input feature, median-imputed the remaining missing entries using SimpleImputer(strategy='median', keep_empty_features=True), and standardized each column using training-partition means and population standard deviations (StandardScaler). Features entirely missing within a training partition were retained with the imputer's zero-fill fallback; this computational fallback did not recode unknown source disease histories as confirmed absence. Constant columns were retained by the scaler. The identical fitted transformations were then applied to the corresponding held-out partition. The classification inputs had 136, 210 and 346 columns for clinical, cytokine and combined models, respectively, including the missingness indicators. These indicators reflect the post-carry-forward feature matrix, including structural visit-column missingness, rather than specifically marking raw below-limit assays.

Sixty-four cytokine measurements originally coded as zero represented concentrations below detection or quantification limits and were treated as unavailable predictors. These entries were not set to true zero concentration or replaced by fractions of a laboratory limit. They followed the past-only carry-forward and training-partition median-imputation procedure described above; no later measurement was used to fill an earlier visit. Matched early-visit LOD/LLOQ information was unavailable for a full censoring-specific analysis. The resulting predictor treatment does not distinguish an unobserved value from a value known to be below a limit, which can affect estimated performance. These 64 source cells should not be assumed to all enter the retained prediction intervals.

## Nested participant grouping and candidate models

Outer validation used the original five participant-grouped partitions generated with StratifiedGroupKFold(shuffle=True, random_state=31) for the three-point outcome. Within each outer training set, three participant-grouped folds were used for hyperparameter selection and calibration. The same original inner and outer partitions were reused for all predictor sets and both outcome thresholds. Thus, the five-point analysis did not independently re-stratify or search for a favorable split. Each participant's intervals remained together at both validation levels, with no participant shared between a corresponding training and held-out partition.

Each candidate preprocessing/classification pipeline was fitted using only its inner training partition. Candidate ranking used the unweighted mean of the three held-out inner AUCs. For logistic regression and SVM, ranking used the decision function; for the two tree-model families, ranking used the positive-class probability. The following 24 settings were evaluated:

| **Family** | **Searched settings** | **Other specified settings** |
| --- | --- | --- |
| L2 logistic regression | C = 0.01, 0.1, 1, 10 | lbfgs, L2 penalty (l1_ratio=0), max_iter = 5,000 |
| Linear SVM | C = 0.01, 0.1, 1 | Linear kernel; probability=False |
| RBF-SVM | C = 0.1, 1, 10 crossed with gamma = scale, 0.01, 0.1 | RBF kernel; probability=False |
| Random forest | Maximum depth = 2 or 4 crossed with minimum leaf size = 5 or 10 | 300 trees; max_features='sqrt'; bootstrap sampling |
| Histogram gradient boosting | Maximum leaves = 3 or 7 crossed with minimum leaf size = 10 or 20 | Learning rate = 0.05; 100 iterations; L2 regularization = 1; early stopping disabled |

Base estimators used class_weight='balanced', recomputed within each training fit, and random_state = 31 where supported. Class weights were derived from interval labels; no inverse-number-of-visits participant weighting was introduced. For gamma = scale, SVC uses the fitted training matrix's feature count and variance to determine gamma. Other estimator defaults were those recorded in the saved scikit-learn 1.9.0 parameter manifest. Training software comprised Python 3.12.14, NumPy 2.4.6, pandas 3.0.3 and scikit-learn 1.9.0.

The best candidate was retained separately within each family and predictor set. Exact ties retained the first setting in the listed order. Outer-fold outcomes were not used for these within-fold choices. The fixed-RBF comparator used C = 1 and gamma = scale with the same median-imputation and calibration pipeline. It was reused when identical to the selected RBF candidate, otherwise fitted separately. The five tuned model families and the fixed-RBF comparator are reported for each predictor set and endpoint.

The final emphasis on tuned RBF-SVM for the manuscript followed exploratory review of these and earlier modeling results. It maintains the original SVM model family and permits a matched-algorithm comparison of predictor sets; it is not an originally prespecified claim that RBF-SVM is the best algorithm.

## Calibration and performance measures

Each independently tuned model and the fixed-RBF comparator underwent sigmoid probability calibration using CalibratedClassifierCV with the same three inner participant-grouped folds and ensemble=False. The complete preprocessing/estimator pipeline was refitted within calibration training folds to generate cross-fitted calibration inputs; the base estimator was also refitted on the full outer training set. The resulting calibration mapping was applied to predictions for that outer held-out set. Calibration used observed training labels without the base estimator's balancing weights. Scikit-learn uses decision_function for calibration when available, including histogram gradient boosting, and otherwise uses probabilities. The method therefore retained scikit-learn's usual calibration input rule even where the inner search convention used a tree probability.

Hyperparameter selection and calibration reused inner labels; calibration did not use a separate selection-independent sample. Outer held-out data were reserved for evaluating the resulting prediction procedure. The principal reported measure was AUC from pooled calibrated out-of-fold probabilities. The unweighted average of the five outer-fold AUCs and the pooled Brier score (mean squared error of predicted probabilities) were reported separately. Brier score is an overall probability-error measure, not a substitute for a calibration intercept, slope or reliability diagram. No such additional calibration assessment, decision-curve analysis or clinical-utility metric was generated in this reanalysis. Different outcome thresholds define different outcomes; their AUCs and Brier scores should not be interpreted as tests of improvement between thresholds.

## Confidence intervals and comparisons

For paired comparisons, the difference was calculated from the same intervals and folds, with combined minus clinical-only as the incremental-discrimination contrast. A participant-level bootstrap drew 62 participants with replacement, retaining all of each drawn participant's intervals, for 2,000 repetitions with NumPy default_rng(31). The same participant draws were used across endpoints, models and comparisons. Score ties received half credit when computing AUC. Percentile 2.5th and 97.5th quantiles, with linear interpolation, defined two-sided 95% confidence intervals. Marginal AUC intervals were calculated from these same draws for the six tuned-RBF results. Single-model intervals were not calculated for the remaining family results; paired-difference intervals are not substituted for them.

Pooled AUC required both outcome classes in the resampled data. Fold-mean AUC additionally required both classes within every original outer fold; invalid replicates were omitted from the corresponding interval calculation. All pooled comparisons had 2,000 valid replicates. Fold-mean comparisons had 2,000 valid replicates for the three-point endpoint and 1,989 for the five-point endpoint. These are conditional intervals for fixed out-of-fold predictions and folds: bootstrap repetitions did not refit, recalibrate or retune models and do not include training, split or model-search uncertainty. Confidence intervals were not adjusted for the multiple exploratory comparisons. An interval crossing zero does not establish equivalence, and overlap between marginal AUC intervals is not a test of the paired difference.

## Scope and interpretation

Performance reflects held-out intervals from participants excluded from fitting under the specified grouping, not a newly performed later-calendar-time or independent-cohort validation. No new visit-4→5 temporal validation, final deployment model or external validation was produced. All results reported here use the specified median-imputation pipeline. Earlier manuscript models and separate exploratory pipelines are not included in this comparison.

Clinical covariate adjustment also does not establish causal cytokine effects or remove medication timing, ascertainment or assay-censoring confounding. Tables S5–S7 report numerical summaries and fold-specific RBF hyperparameters. Workbook Tables S4A–S4C retain aggregate outputs for the reported individual-family models.

# Supplementary tables

## Table S1 Primary GEE omnibus interactions meeting the FDR threshold

| **Outcome** | **Cytokine** | **Participants / records** | **Raw P** | **BH-adjusted P** |
| --- | --- | --- | --- | --- |
| UPDRS-III | IL-17 | 59 / 218 | 2.25465e-05 | 0.00114138 |
| tremor | IL-12 | 59 / 220 | 0.00231392 | 0.0347088 |
| akinetic-rigid | IL-17 | 59 / 218 | 1.79551e-07 | 1.88528e-05 |
| akinetic-rigid | IL-23 | 59 / 220 | 0.00226387 | 0.0347088 |
| PIGD | IL-4 | 59 / 220 | 0.00300251 | 0.0394079 |
| PIGD | IL-12 | 59 / 220 | 9.59647e-05 | 0.00251907 |
| MoCA | IL-1β | 59 / 206 | 3.26108e-05 | 0.00114138 |
| MoCA | IL-15 | 59 / 206 | 0.00122189 | 0.0256597 |

Each row is a four-degree-of-freedom joint cytokine-by-visit test, with correction across all 105 tests. Models include baseline disease duration. A joint test has no single effect coefficient or confidence interval. Constituent effects and pointwise confidence intervals for every tested pair are in workbook Tables S3B and S3C.

## Table S2 GEE sensitivity analyses

| **Model variant** | **Raw P < 0.05** | **BH-adjusted P < 0.05** | **Primary findings retained** |
| --- | --- | --- | --- |
| Full adjustment, log₂, exchangeable | 28 | 8 | 8 |
| Age/sex only, same complete records | 32 | 14 | 8 |
| Full adjustment, log₂, independence | 26 | 7 | 6 |
| Full adjustment, raw concentration | 45 | 30 | 6 |
| Full adjustment, log₂, exclude documented steroid/NSAID visits | 31 | 7 | 5 |

Each variant has a separate family of 105 omnibus interaction tests. Full adjustment includes baseline disease duration. Retention counts describe the 8 primary pairs, not the total number of discoveries in that variant. Full pair-level values are in workbook Tables S3D and S3H.

## Tables S3A–S3I Complete GEE results in the companion workbook

The full numerical tables are provided in Supplementary_Statistical_Tables.xlsx. The following entries describe its worksheets.

**S3A Omnibus105.** All 105 primary four-degree-of-freedom interaction tests and the separate five-degree-of-freedom global cytokine tests, each with raw P and its own 105-test BH-adjusted P. Participant counts, observation counts, counts at each visit, working correlations and fit diagnostics are included. This sheet contains joint test statistics, not a single coefficient or confidence interval for each joint hypothesis.

**S3B Interactions420.** Every primary-model interaction estimate comparing a Visit 2–5 cytokine–score slope with its Visit 1 slope, with robust standard error, pointwise 95% CI, raw P, BH-adjusted P across 420 contrasts and sample sizes. Units are clinical-score units per cytokine doubling.

**S3C VisitSlopes525.** All primary-model total slopes at Visits 1–5, with robust standard error, covariance-derived pointwise 95% CI, raw P and BH-adjusted P across 525 slopes. The 105 Visit 1 rows explicitly provide the main cytokine coefficients. These slopes are distinct from the interaction contrasts in S3B.

**S3D AllModels525.** Omnibus and global tests for all five model variants, including the primary models. Each variant has separate 105-test families for interaction and global hypotheses. Rows include full sample-size and convergence information.

**S3E AllInteractions2100.** All 420 interaction coefficients for each of the five variants, with robust SE, pointwise 95% CI, raw P and a 420-test BH correction within each variant.

**S3F AllSlopes2625.** All 525 total slopes for each of the five variants, with robust SE, pointwise 95% CI, raw P and a 525-test BH correction within each variant. The raw-concentration variant uses concentration units; the others use cytokine doublings.

**S3G Missingness105.** Model-specific candidate, paired-outcome/cytokine and complete-record counts, together with missingness counts for the outcome, cytokine and covariates. Counts across missing variables overlap and should not be summed. Empty cells in variable-specific columns mean that the variable was not the outcome or cytokine for that row, not zero missingness.

**S3H PrimarySensitivity.** The 8 primary pairs and their omnibus BH-adjusted P values in every variant. This is a selected summary; S3D contains all pairs, including additional findings in sensitivity variants.

**S3I InfluenceSummary.** Aggregate summary of 472 participant-omission fits across the 8 primary pairs, including the number of valid fits, raw-P ranges and maximum standardized coefficient change. No BH correction was applied to these diagnostics.

Participant-level omission records are not included in the companion workbook; S3I provides the aggregate summary of all 472 diagnostic fits without individual participant codes.

## Tables S4A–S4C Results for the reported individual-family models in the companion workbook

**S4A ModelSummary.** The 36 combinations of two outcome thresholds, six strategies and three predictor sets, including pooled AUC, fold-mean AUC and Brier score.

**S4B PairedComparisons.** The 44 comparison rows for the reported individual-family models, comprising pooled and fold-mean AUC differences, percentile confidence intervals and valid bootstrap counts. These include combined-minus-clinical comparisons and comparisons against the fixed RBF model.

**S4C MarginalAUC_CI.** The six marginal pooled-AUC intervals for the tuned-RBF results. No marginal intervals were calculated for the other family models.

The ReadMe sheet explains source labels and missing cells. Worksheet names use underscores, for example S3A_Omnibus105 and S4A_ModelSummary. Full numeric precision is preserved in the workbook; displayed values in this document are rounded.

## Table S5 Reported individual-family models for both outcomes and predictor sets

| **Outcome** | **Strategy** | **Predictors** | **Pooled AUC** | **Fold-mean AUC** | **Brier score** |
| --- | --- | --- | --- | --- | --- |
| ≥3 points | L2 logistic regression | Clinical only | 0.714 | 0.726 | 0.209 |
| ≥3 points | L2 logistic regression | Cytokine only | 0.686 | 0.692 | 0.219 |
| ≥3 points | L2 logistic regression | Combined | 0.702 | 0.720 | 0.209 |
| ≥3 points | Linear SVM | Clinical only | 0.723 | 0.730 | 0.208 |
| ≥3 points | Linear SVM | Cytokine only | 0.687 | 0.683 | 0.222 |
| ≥3 points | Linear SVM | Combined | 0.687 | 0.704 | 0.216 |
| ≥3 points | Tuned RBF-SVM | Clinical only | 0.733 | 0.755 | 0.207 |
| ≥3 points | Tuned RBF-SVM | Cytokine only | 0.722 | 0.749 | 0.209 |
| ≥3 points | Tuned RBF-SVM | Combined | 0.784 | 0.802 | 0.186 |
| ≥3 points | Random forest | Clinical only | 0.740 | 0.753 | 0.197 |
| ≥3 points | Random forest | Cytokine only | 0.685 | 0.705 | 0.219 |
| ≥3 points | Random forest | Combined | 0.739 | 0.765 | 0.202 |
| ≥3 points | Histogram gradient boosting | Clinical only | 0.723 | 0.748 | 0.201 |
| ≥3 points | Histogram gradient boosting | Cytokine only | 0.625 | 0.640 | 0.234 |
| ≥3 points | Histogram gradient boosting | Combined | 0.652 | 0.680 | 0.228 |
| ≥3 points | Fixed RBF-SVM | Clinical only | 0.728 | 0.751 | 0.193 |
| ≥3 points | Fixed RBF-SVM | Cytokine only | 0.750 | 0.765 | 0.203 |
| ≥3 points | Fixed RBF-SVM | Combined | 0.770 | 0.779 | 0.188 |
| ≥5 points | L2 logistic regression | Clinical only | 0.758 | 0.786 | 0.188 |
| ≥5 points | L2 logistic regression | Cytokine only | 0.690 | 0.702 | 0.210 |
| ≥5 points | L2 logistic regression | Combined | 0.735 | 0.764 | 0.193 |
| ≥5 points | Linear SVM | Clinical only | 0.759 | 0.793 | 0.189 |
| ≥5 points | Linear SVM | Cytokine only | 0.682 | 0.683 | 0.210 |
| ≥5 points | Linear SVM | Combined | 0.712 | 0.748 | 0.193 |
| ≥5 points | Tuned RBF-SVM | Clinical only | 0.743 | 0.795 | 0.190 |
| ≥5 points | Tuned RBF-SVM | Cytokine only | 0.675 | 0.663 | 0.192 |
| ≥5 points | Tuned RBF-SVM | Combined | 0.790 | 0.805 | 0.169 |
| ≥5 points | Random forest | Clinical only | 0.783 | 0.815 | 0.170 |
| ≥5 points | Random forest | Cytokine only | 0.735 | 0.756 | 0.182 |
| ≥5 points | Random forest | Combined | 0.771 | 0.790 | 0.173 |
| ≥5 points | Histogram gradient boosting | Clinical only | 0.802 | 0.826 | 0.166 |
| ≥5 points | Histogram gradient boosting | Cytokine only | 0.702 | 0.711 | 0.195 |
| ≥5 points | Histogram gradient boosting | Combined | 0.762 | 0.773 | 0.179 |
| ≥5 points | Fixed RBF-SVM | Clinical only | 0.765 | 0.804 | 0.174 |
| ≥5 points | Fixed RBF-SVM | Cytokine only | 0.772 | 0.796 | 0.180 |
| ≥5 points | Fixed RBF-SVM | Combined | 0.791 | 0.818 | 0.163 |

Pooled AUC and Brier score use calibrated out-of-fold probabilities. Fold-mean AUC is the unweighted mean over the five outer folds. Lower Brier score indicates lower mean squared probability error; it is not a calibration-specific statistic. Results for each reported individual-family model are shown for all predictor sets and both thresholds. The fixed-RBF row uses the same median-imputation pipeline as the tuned models.

## Table S6 Paired Combined−Clinical AUC differences for the reported individual-family models

| **Outcome** | **Strategy** | **Pooled ΔAUC (95% CI)** | **Fold-mean ΔAUC (95% CI)** | **Valid bootstrap pooled / fold-mean** |
| --- | --- | --- | --- | --- |
| ≥3 points | L2 logistic regression | -0.012 (-0.078 to 0.054) | -0.005 (-0.069 to 0.070) | 2000 / 2000 |
| ≥3 points | Linear SVM | -0.036 (-0.105 to 0.028) | -0.027 (-0.093 to 0.049) | 2000 / 2000 |
| ≥3 points | Tuned RBF-SVM | +0.051 (-0.009 to 0.112) | +0.047 (-0.033 to 0.135) | 2000 / 2000 |
| ≥3 points | Random forest | -0.002 (-0.054 to 0.049) | +0.011 (-0.051 to 0.070) | 2000 / 2000 |
| ≥3 points | Histogram gradient boosting | -0.070 (-0.145 to 0.008) | -0.067 (-0.149 to 0.018) | 2000 / 2000 |
| ≥3 points | Fixed RBF-SVM | +0.043 (-0.013 to 0.098) | +0.028 (-0.031 to 0.088) | 2000 / 2000 |
| ≥5 points | L2 logistic regression | -0.023 (-0.088 to 0.041) | -0.022 (-0.078 to 0.049) | 2000 / 1989 |
| ≥5 points | Linear SVM | -0.047 (-0.126 to 0.035) | -0.044 (-0.119 to 0.051) | 2000 / 1989 |
| ≥5 points | Tuned RBF-SVM | +0.047 (-0.023 to 0.112) | +0.010 (-0.064 to 0.083) | 2000 / 1989 |
| ≥5 points | Random forest | -0.012 (-0.076 to 0.052) | -0.025 (-0.093 to 0.044) | 2000 / 1989 |
| ≥5 points | Histogram gradient boosting | -0.040 (-0.113 to 0.030) | -0.053 (-0.136 to 0.027) | 2000 / 1989 |
| ≥5 points | Fixed RBF-SVM | +0.026 (-0.029 to 0.082) | +0.014 (-0.043 to 0.074) | 2000 / 1989 |

All differences compare the combined and clinical-only models of the named strategy on matched intervals and partitions. Confidence intervals are unadjusted and conditional on the fixed predictions/folds; an interval containing zero does not establish equivalence.

## Table S7 RBF hyperparameters in each outer training set

| **Outcome** | **Predictors** | **Outer fold** | **RBF C** | **RBF gamma** |
| --- | --- | --- | --- | --- |
| ≥3 points | Clinical only | 1 | 10 | 0.01 |
| ≥3 points | Clinical only | 2 | 1 | 0.01 |
| ≥3 points | Clinical only | 3 | 0.1 | scale |
| ≥3 points | Clinical only | 4 | 1 | 0.01 |
| ≥3 points | Clinical only | 5 | 10 | 0.01 |
| ≥3 points | Cytokine only | 1 | 1 | scale |
| ≥3 points | Cytokine only | 2 | 1 | 0.01 |
| ≥3 points | Cytokine only | 3 | 10 | 0.01 |
| ≥3 points | Cytokine only | 4 | 1 | 0.1 |
| ≥3 points | Cytokine only | 5 | 1 | 0.01 |
| ≥3 points | Combined | 1 | 10 | 0.01 |
| ≥3 points | Combined | 2 | 1 | 0.01 |
| ≥3 points | Combined | 3 | 10 | 0.01 |
| ≥3 points | Combined | 4 | 1 | scale |
| ≥3 points | Combined | 5 | 10 | scale |
| ≥5 points | Clinical only | 1 | 1 | scale |
| ≥5 points | Clinical only | 2 | 1 | 0.01 |
| ≥5 points | Clinical only | 3 | 0.1 | 0.01 |
| ≥5 points | Clinical only | 4 | 1 | 0.01 |
| ≥5 points | Clinical only | 5 | 1 | scale |
| ≥5 points | Cytokine only | 1 | 1 | scale |
| ≥5 points | Cytokine only | 2 | 1 | 0.1 |
| ≥5 points | Cytokine only | 3 | 10 | 0.01 |
| ≥5 points | Cytokine only | 4 | 0.1 | 0.1 |
| ≥5 points | Cytokine only | 5 | 0.1 | 0.1 |
| ≥5 points | Combined | 1 | 1 | scale |
| ≥5 points | Combined | 2 | 1 | 0.01 |
| ≥5 points | Combined | 3 | 10 | scale |
| ≥5 points | Combined | 4 | 1 | scale |
| ≥5 points | Combined | 5 | 10 | scale |

Each row identifies RBF hyperparameter tuning using only that outer training set. RBF parameters were selected independently for each predictor set and outcome. Full parameter manifests and selected inner-validation scores are available with the analysis code from the corresponding author upon reasonable request.

# Supplementary figures

## Figure S1 Characterization of plasma EV-enriched preparations

Representative characterization images retained from the original study: (A) nanoparticle tracking analysis showing the particle-size distribution; (B) Western blot analysis of CD63, CD9, CD81 and TSG101; and (C) transmission electron microscopy. These measurements support the presence of EV-containing material. They do not establish a purified exosome population or determine whether the assayed cytokines were intraluminal, membrane-associated or co-isolated with non-vesicular material. The original experimental image is reproduced without alteration of its analytical content.
